# Treatment of Coexisting Chronic Neutrophilic Leukemia and Light Chain Multiple Myeloma with Hydroxyurea, Bortezomib, and Dexamethasone

**DOI:** 10.1155/2014/869395

**Published:** 2014-07-17

**Authors:** Evelyn Taiwo, Huiying Wang, Robert Lewis

**Affiliations:** ^1^Kings County Hospital Center, 451 Clarkson Avenue, Brooklyn, NY 11203, USA; ^2^State University of New York, Downstate, Brooklyn, NY, USA

## Abstract

A 63-year-old female was incidentally found to have leukocytosis and referred to the hematology service for evaluation. Complete blood count (CBC) revealed neutrophilia with band predominance and mild thrombocytopenia. Peripheral blood flow cytometry was unremarkable without any evidence of lymphoproliferative disorder or myeloblasts. Bone marrow aspiration and biopsy revealed a markedly hypercellular marrow with myeloid lineage predominance and approximately 10% plasma cells. The monoclonal gammopathy was determined as lambda light chain with a kappa/lambda ratio of 0.06. Cytogenetics revealed normal karyotype, JAK2 kinase was negative, and rearrangement of BCR-ABL1, PDGFRA, PDGFRB, and FGFR1 was negative. The patient was diagnosed with chronic neutrophilic leukemia (CNL) associated with light chain multiple myeloma, complicated by a subdural hemorrhage. She was treated with hydroxyurea and bortezomib/dexamethasone and had complete response with normalization of CBC and kappa/lambda ratio. To the best of our knowledge, we report the first case of chronic neutrophilic leukemia and multiple myeloma treated with bortezomib/dexamethasone.

## 1. Background

Chronic neutrophilic leukemia (CNL) is a rare myeloproliferative neoplasm and a diagnosis is only made in the absence of reactive neutrophilia, myeloproliferative neoplasm (MPN), and myelodysplastic syndrome (MDS) or overlap of MDS/MPN. Absence of BCR-ABL1, PDGFRA, PDGFRB, and FGFR1 rearrangements is also minimal diagnostic requirements for CNL [1]. According to the 2008 World Health Organization (WHO), diagnostic criteria for CNL are leukocytosis >25 × 10^9^/L; >80% are segmented neutrophils; and <10% are immature granulocytes with the absence of granulocytic dysplasia, monocytosis, eosinophilia, and basophilia [1]. Additional clinicopathologic characteristics of CNL include splenomegaly, elevated vitamin B12 level, and neutrophilic leukocytosis characterized by toxic granulation and Döhle bodies [1]. Intracranial hemorrhage likely due to platelet dysfunction with leukemic infiltration and destruction of vessels [2, 3], blast transformation, and treatment related toxicity were the most common causes of death in these patients [4].

Even rarer than CNL is the coexistence of the disease with multiple myeloma. This rare phenomenon has been reported in the literature with this subset of patients presenting with a monoclonal gammopathy associated with *λ* light chain excess [5]. Cytogenetic abnormalities are absent in these reported cases and it remains unclear if the neutrophilic leukocytosis is a result of a myeloproliferative process or a leukemoid response to the monoclonal gammopathy.

The previously reported cases of the coexistence of CNL and multiple myeloma have primarily focused on the presence of this phenomenon and the possible nature of the relationship between the two disease processes. Management has not been addressed in these discussions, and when reported, the patients were primarily treated with cytoreductive therapy. Most of the patients in the reported cases were treated before the approval of bortezomib for treatment of multiple myeloma and the medication was not included in any treatment regimen. We report a case of CNL associated with multiple myeloma, treated with hydroxyurea, bortezomib, and dexamethasone, with complete resolution of leukocytosis and monoclonal gammopathy.

## 2. Case Presentation

A 63-year-old African American female with history of hypertension, type II diabetes, and hyperlipidemia was referred to the hematology service for newly discovered leukocytosis. CBC at her initial hematology clinic revealed a white blood count (WBC) 65,590/uL (69% segmented neutrophils, 22% bands, 4% lymphocytes, 2% monocytes, 1% eosinophils, 1% metamyelocytes, and 1% myelocytes), hemoglobin 15 g/dL, and platelets 95,000/uL.

**Figure 1:**
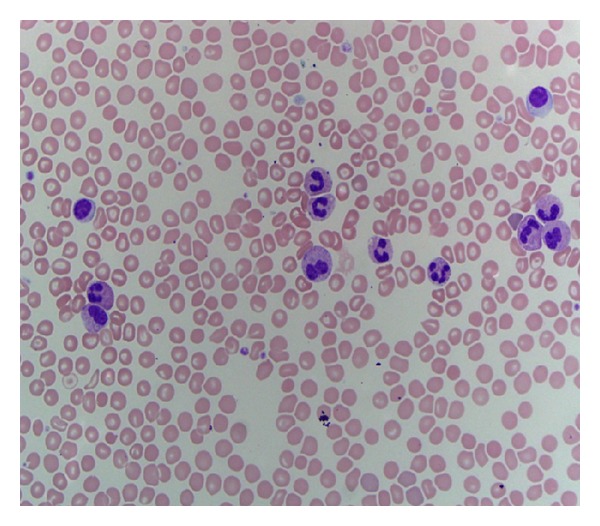
Blood smear showing segmented neutrophils with arrow pointing at Döhle bodies.

The patient reported a 10 lb weight loss over an 8-month period but otherwise was without any B symptoms. Her physical examination was essentially unremarkable without evidence of hepatosplenomegaly. Blood smear was remarkable for marked leukocytosis predominantly composed of mildly left shifted neutrophils with mild cytoplasmic toxic granules and Döhle bodies (Figure 1).

**Figure 2:**
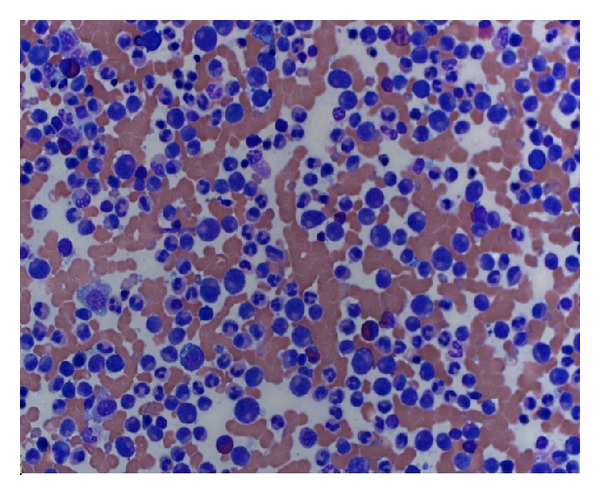
Bone marrow aspiration reveals predominance of myeloid lineage.

**Figure 3:**
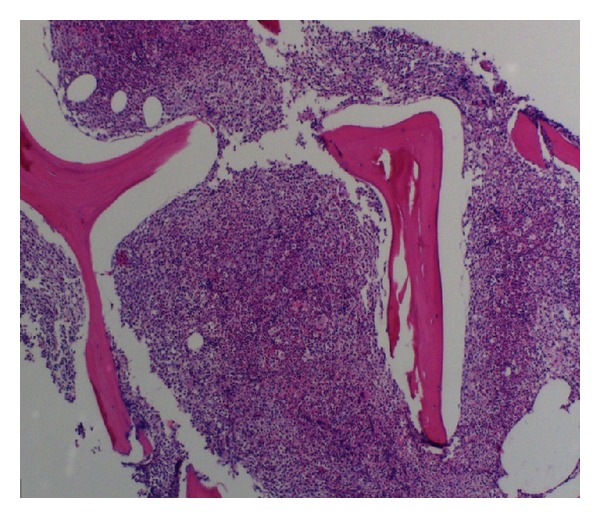
Bone marrow biopsy reveals a markedly hypercellular marrow.

Additional testing including Jak2 kinase, BCR-ABR1, PDGFRA, PDGFRB, and FGFR1 rearrangement was negative, and CT scans of the chest, abdomen, and pelvis were negative for lymphadenopathy or splenomegaly. Bone marrow aspiration and biopsy revealed a markedly hypercellular marrow with predominance of myeloid lineage (Figures 2 and 3), mild reticulin fibrosis, and approximately 10% plasma cells with reversed kappa/lambda ratio. Immunohistochemistry showed rare CD117 and CD34 blasts. CD138 revealed approximately 10% plasma cells predominantly expressing lambda light chains. 83% of the cells were granulocytic precursors in varying stages of maturation, estimated M : E ratio 6 : 1.

Serum protein electrophoresis was normal, kappa light chain was 17.1 g/L, and lambda light chain was 276.9 g/L, with a ratio of 0.06. Albumin, creatinine, and calcium were within normal limits and skeletal survey was negative for lytic lesions. A diagnosis of smoldering lambda light chain multiple myeloma was made based on the presence of 10% plasma cells in the bone marrow, the increased free lambda light chains, and the abnormal kappa/lambda light chain ratio.

Approximately 3 weeks after the diagnosis of multiple myeloma, the patient's thrombocytopenia and leukocytosis worsened and hydroxyurea 1 gram daily was initiated. 14 days after initiation of treatment, the patient presented to the hospital with a severe headache with associated nausea and vomiting. CT scan of the brain revealed an acute subdural hematoma (aSDH) with mass effect on the left lateral ventricle and midline shift to the right. CBC at the time of presentation with the aSDH revealed WBC 80,320/uL, hgb 12.5 g/dL, and platelets 109,000/uL. Platelet transfusion was given and the patient was managed conservatively with dexamethasone and serial CT scans, until scans revealed resorption of the subdural hematoma.

The patient remained on single therapy with hydroxyurea for 4 weeks with resolution of thrombocytopenia. Hydroxyurea dose was not increased due to platelet response to treatment. However, due to the persistent leukocytosis, bortezomib and dexamethasone were added to treat the lambda light chain multiple myeloma. The patient received bortezomib 1.3 mg/m^2^ on days 1, 4, 8, and 11 every 3 weeks, and dexamethasone 40 mg weekly. The improvement of leukocytosis led to discontinuation of hydroxyurea 2 months after initiating bortezomib/dexamethasone. The patient was treated with 6 cycles of therapy, with normalization of the CBC and free light chains. The patient remains asymptomatic and remains off treatment 12 months after presentation.

## 3. Discussion

The coexistence of chronic neutrophilic leukemia and multiple myeloma is a well-reported phenomenon with at least 12 cases in the literature. However, it remains unclear whether the neutrophilic leukocytosis is a leukemoid response to the underlying monoclonal gammopathy, or if the presence of the two diseases represents a real entity. Some investigators have concluded that the leukocytosis is in response to the myeloma because monoclonal B-cell clones in myeloma can produce cytokines which are able to activate stromal cells to produce IL-6, IL-7, and IL-11 to stimulate T lymphocytes to produce IL-3 and GM-CSF [6]. Others have argued that the presence of pronounced organ infiltration by neutrophils in reported cases is strong evidence against a leukemoid state [5].

The recent discovery of mutations in the receptor for colony-stimulating factor 3 (CSF3R; GCSFR), a commercially available mutation of which 50–60% of patients with CNL have been reported to harbor [4], may improve our ability to determine the clonality of the neutrophils in CNL and help confirm or refute the coexistence of CNL and multiple myeloma as a real phenomenon.

In our case, the diagnosis of multiple myeloma was made based on the presence of 10% plasma cells in the bone marrow and the presence of increased lambda light chains. Since our patient did not have any evidence of end organ damage such as bony lesions, renal failure, or hypercalcemia, a diagnosis of smoldering multiple myeloma is likely a more befitting diagnosis. However, the patient's persistent leukocytosis and lack of response to cytoreductive therapy with hydroxyurea prompted a different approach to treatment and consideration of treatment for multiple myeloma. Since some investigators have suggested that the leukocytosis in these types of cases could be a leukemoid response to the plasma cell neoplasm, the decision was made to treat our patient as symptomatic multiple myeloma.

In our patient, the resolution of leukocytosis with treatment for multiple myeloma suggests a reactive leukocytosis; however, the patient's clinical course particularly the development of acute subdural hemorrhage cannot simply be explained as a leukemoid reaction. To the best of our knowledge, there are no documented cases of reactive leukocytosis causing intracranial hemorrhages. However, intracranial hemorrhage is a common cause of death in patients with CNL. The presence of toxic granulation and Döhle bodies is consistent with CNL, and, furthermore, our patient fulfilled the diagnostic criteria except for the absence of splenomegaly. Isolated cases of CNL without splenomegaly have been reported; therefore the absence of splenomegaly does not rule out a diagnosis in this patient [7, 8].

The relationship between CNL and multiple myeloma remains unclear without a consensus on the management of this rare phenomenon. As stated earlier, the complete response of the neutrophilia to multiple myeloma treatment is suggestive of a reactive process, but the patient's clinical course was not consistent with a reactive process. Until further studies establish the clonality of the neutrophilic leukocytosis, a primary diagnosis of CNL versus a leukemoid reaction will remain difficult to distinguish, and treating the underlying monoclonal gammopathy in addition to cytoreductive therapy should be considered.
